# Neurosurgery and artificial intelligence

**DOI:** 10.3934/Neuroscience.2021025

**Published:** 2021-08-06

**Authors:** Mohammad Mofatteh

**Affiliations:** 1 Sir William Dunn School of Pathology, Medical Sciences Division, University of Oxford, South Parks Road, Oxford OX1 3RE, United Kingdom; 2 Lincoln College, University of Oxford, Turl Street, Oxford OX1 3DR, United Kingdom

**Keywords:** neurosurgery, neurological surgery, brain surgery, spine surgery, artificial intelligence, machine learning, deep learning, nervous system, diagnosis, prognosis, global health

## Abstract

Neurosurgeons receive extensive and lengthy training to equip themselves with various technical skills, and neurosurgery require a great deal of pre-, intra- and postoperative clinical data collection, decision making, care and recovery. The last decade has seen a significant increase in the importance of artificial intelligence (AI) in neurosurgery. AI can provide a great promise in neurosurgery by complementing neurosurgeons' skills to provide the best possible interventional and noninterventional care for patients by enhancing diagnostic and prognostic outcomes in clinical treatment and help neurosurgeons with decision making during surgical interventions to improve patient outcomes. Furthermore, AI is playing a pivotal role in the production, processing and storage of clinical and experimental data. AI usage in neurosurgery can also reduce the costs associated with surgical care and provide high-quality healthcare to a broader population. Additionally, AI and neurosurgery can build a symbiotic relationship where AI helps to push the boundaries of neurosurgery, and neurosurgery can help AI to develop better and more robust algorithms. This review explores the role of AI in interventional and noninterventional aspects of neurosurgery during pre-, intra- and postoperative care, such as diagnosis, clinical decision making, surgical operation, prognosis, data acquisition, and research within the neurosurgical arena.

## 1. Introduction

Neurosurgery is a demanding profession. Successful neurosurgeons require extensive training, stamina, a high degree of manual dexterity, excellent hand-eye coordination, intelligent decision making, leadership and organisational skills, compassion, communication skills and teamwork [1]. The boundaries and outcomes of surgeries are in part limited by the skills of the operating surgeons [2], rendering variabilities in the outcomes and experience of patients undergoing the same operation within different settings. While successful operations can benefit patients, errors can have undesired outcomes and, sometimes, harmful consequences. For instance, about a quarter of medical errors occurring in neurosurgery are technical errors related to the surgical procedures [3] that can be prevented, emphasising the importance of pragmatic steps to improve the successful outcome of neurosurgical interventions and reducing associated errors to deliver the best possible care for patients. Recent advances in technology have bridged the gap between humans and machines and have enabled computers to mimic, and even outperform, natural human intelligence to create what is called “artificial intelligence” (AI).

Since Kwoh and colleagues used the first robotic procedure in the modern era for computerised tomography (CT)-guided stereotactic brain surgery in 1988 [4], neurosurgeons have always been at the forefront of using cutting-edge technologies to deliver the best possible care for their patients. Noninvasive visualisation techniques such as CT scanning, magnetic resonance imaging (MRI) combined with image guiding, stereotactic surgery, and electrical stimulation have been essential components of neurosurgery within and out of operating rooms [5],[6]. Multiple technologically advanced methods such as self-positioning microscopes and endoscopes or automatic imaging guidance procedures have been used to improve patient outcomes and to reduce the procedural errors associated with neurosurgery [7]–[9]. As the overall life expectancy and the global population levels are rising [10]–[12], the demand for healthcare increases; however, the capacity of healthcare systems cannot address the surge in demand by solely relying on the human capital. The “crisis in human resources” is one of the most significant challenges of the healthcare sector in the recent era [13]. Furthermore, the ease of access to various healthcare services have caused a huge surge in the production and storage of clinical data such as imaging, genomics, and health monitoring [14]. For example, 80% of about 15 million global cancer diagnoses require surgery [15], which, in turn, each requires pre-, intra-, and postoperative clinical data collection, processing, interpretation and storage by human.

More than 13.8 million patients undergo a neurosurgical procedure globally every year [16]. Although it is estimated that there has been a global growth in the number of neurosurgeons to about 50,000 neurosurgeons currently [17], more than 5 million individuals in low- and middle-income countries with treatable neurosurgical conditions remain untreated annually [16], creating a huge gap between essential neurosurgery required and current neurosurgeries performed. Approximately 23,000 neurosurgeons are required to address global deficiencies in neurosurgery, particularly in low- and middle-income countries [16]. However, training neurosurgeons is competitive, lengthy, expensive and requires highly committed mentors and advanced surgical equipment [18]–[21]. These statistics are concerning and require sustainable and thorough solutions to address global health concerns.

Machines, algorithms and AI do not face mental and physical fatigue and can function 24/7, therefore, have a higher safety standard compared to humans. Additionally, machines have a greater capacity to learn and identify patterns that are not obvious to humans [22], thereby demonstrating hidden connections which are hard to discern [23]. Interpretation of clinical data, especially radiological images, can be subjective and qualitative [24],[25] causing heterogeneity of diagnoses among physicians and, in some cases, poor prognosis. AI can benefit neurosurgeons by reducing errors in the surgical arena, reducing costs associated with diagnosis, treatment and prognosis, expanding access to high-quality medical care as well as providing patients with increased autonomy in their own decision-making processes.

**Figure 1.**
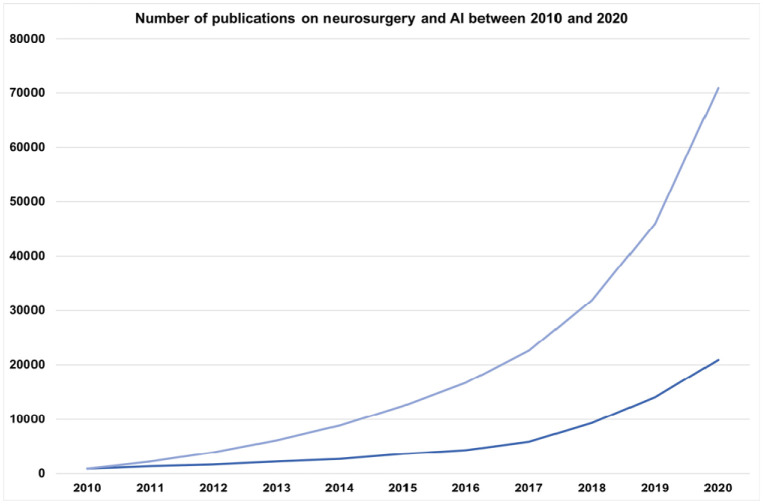
Absolute and the cumulative number of publications involved neurosurgery and artificial intelligence in their title or abstract over the past decade. The representative data was gathered from the database PubMed using neurosurgery OR neurological surgery OR brain surgery AND artificial intelligence OR machine learning OR deep learning search function in the title or abstract from 2010–2020.

The use of technology, especially AI and robotics, in medicine and surgical interventions has been increasing over the past decade [26]–[28]. A search in the database PubMed showed a sustained increasing trend in the number of publications involving neurosurgery and artificial intelligence over the past decade (Figure 1).

While AI can benefit multiple parties such as surgeons, healthcare workers, the overall healthcare system and patients [29], in this review, I mainly focus on the benefits of AI on neurosurgeons while briefly mentioning the wider aspect of AI on the healthcare system.

## 2. Artificial intelligence and neurosurgery

**Figure 2.**
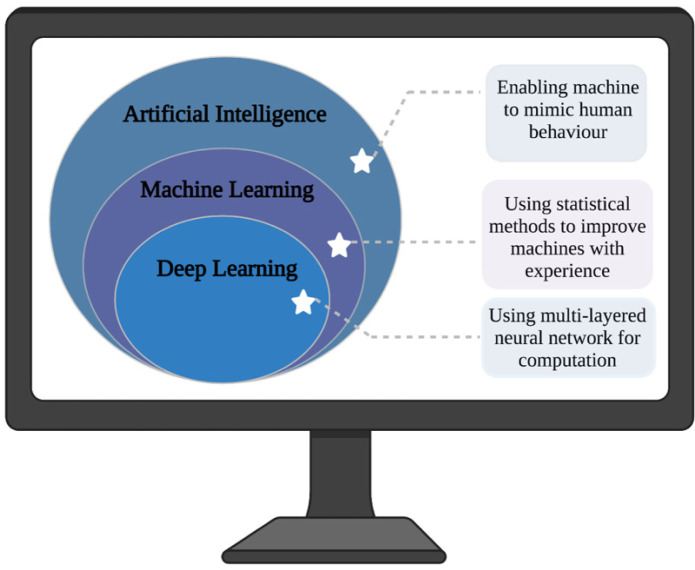
An overview of the relationship between artificial intelligence, machine learning and deep learning. AI aims to mimic the intelligent behaviour of humans. ML as a branch of AI uses statistics and computer sciences to improve the performance of machines as the experience accumulates. DL uses multi-layered neural networks to learn computation. The figure was made using Biorender.

AI, machine learning (ML) and deep learning (DL) have the potential to transform neurosurgery. AI aims to simulate the behaviour of intelligent beings in computers, whereas ML as a subdomain of AI combines computer science and statistics to enable computers to learn patterns by direct studying of data through experience, autonomous of external programming [30],[31] (Figure 2). Our brain changes as we grow, and so does ML as it trains. In its essence, ML is acting similar to medical students and resident doctors to learn rules from data and to apply general rules to various patients in each case with one chief difference- doing these on a huge scale with an enormous amount of data [22] (Figure 2). ML in medical sciences mainly uses supervised learning through training algorithms such as logistic regression, support vector machine and random forests [25].

**Figure 3.**
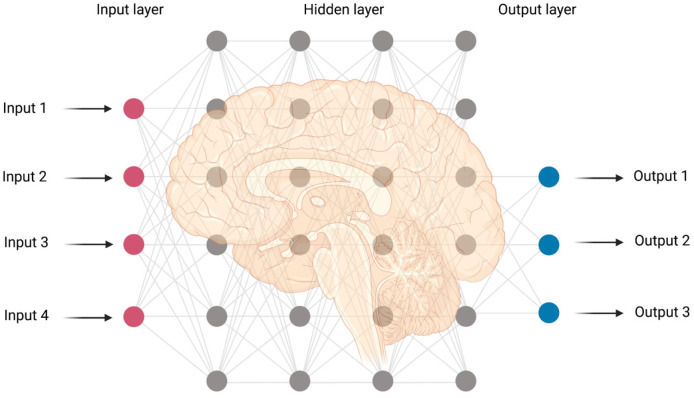
An overview of multilayer perception in the context of the artificial neural network of deep learning model with multiple interconnected layers. The figure was made using Biorender.

AI can improve the accuracy of diagnosis and treatment in neurosurgery and provide neurosurgeons with effective and efficient tools in a timely manner during pre-, intra- and postoperative care. AI can spot subtle abnormalities and malformations from neuroradiological images and clinical data which are not discernible to trained eyes. Deep learning as a subset of ML is based on neural networks, which include multiple layers of learning algorithms [32] (Figure 3).

Patients can experience highly variable outcomes for the same neurosurgical treatments depending on various underlying reasons such as the experience of the operating surgeon, variations in the operating approach and a lack of clear national or international guidelines for common treatments, financial reasons, annual case volume, geographical location and the nature of the practice (academic, private, etc.) [33]–[37]. It is worth mentioning that other factors influencing the patient outcome in neurosurgical interventions can be patient-specific variations such as age, sex, comorbidities, alcohol and tobacco consumption, body mass index, psychological conditions, and etc. [37]. Considering all these variables to reach a decision for the best surgical intervention can be beyond the capacity of a human being. AI can reduce variations in patient outcome by reducing the heterogeneity of the care via providing guidelines to create consensus among neurosurgeons on surgical interventions, thereby improving prognosis and reducing costs.

**Figure 4.**
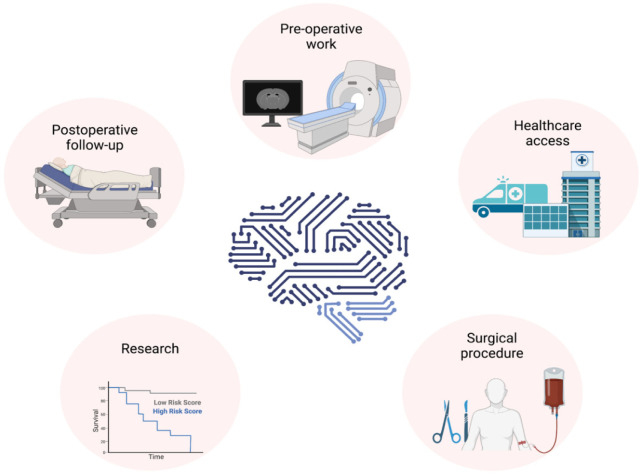
An overview of the role of AI in neurosurgery. AI can help the neurosurgery in pre-operative work, intra-operative surgical procedures, postoperative follow-up, improving clinical research and expanding access to healthcare. The figure is providing an overview, and it is not an exhaustive list. The figure was made using Biorender.

AI can improve the patient outcomes in neurosurgery in pre-, intra- and postoperative domains as well as neurosurgical research, training and expanding access to high-quality treatments (Figure 4).

## 3. The role of AI in pre-, intra- and postoperative phases of neurosurgery

In the pre-operative phase of neurosurgery, AI can assist surgeons in diagnosing the condition, selecting patients for the right treatment, and helping patients to make the right decisions [31]. In the intra-operative phase of neurosurgery, AI can enhance the performance of surgeons and reduce errors associated with neurosurgery. In postoperative care, AI can predict prognosis, identify potential postoperative complications and track data for better aftercare and recovery. As such, by making better predictions in the postoperative phase, pre-operative planning can be enhanced to improve patient care and reduce the associated costs. For example, ML can be used for classification, regression and clustering to analyse large data sets, identify risk factors and predict surgical complications such as cardiac complications, wound complications and mortality among patients following a cervical discectomy and posterior lumbar spine fusion [38]–[40]. Research in neurosurgery can benefit from AI by helping researchers to collect, analyse, process, and disseminate data. Since available financial means can be an obstacle for delivering the best possible treatment, AI can facilitate access to high-quality care in less developed areas through teleoperation. I am going to discuss each of these domains below.

High-resolution and ubiquitous radiological imaging combined with electrophysiological data are methods of choice that have provided neurosurgeons with unprecedented and noninvasive intracranial access. Manual decision making in neurosurgical medicine requires neurosurgeons to study, retain, analyse and interpret a large quantity of complicated and dynamic data. Neurosurgeons usually rely on their experience and clinical evidence to make a decision and provide a prognosis [41],[42]. Regardless of how well-trained or experienced a neurosurgeon is, manual handling of such information can be, at its very least, challenging for the human capacity. Furthermore, the sheer volume of data combined with the urge to make a rapid and accurate diagnosis, the presence of atypical cases, and lack of access to trained radiologists can pose bottlenecks for the accurate and timely diagnosis of different conditions manually by trained physicians and be resource-, time-, and labour intensive. As such, errors and discrepancies can be as common as 3–5% in radiology [43]. In addition, time is of crucial importance in neurosurgical interventions, and reducing the door-to-needle times for emergency neurosurgical interventions has been a goal for many clinical settings [44].

Some challenges in neurosurgical care can be circumvented by computer-assisted diagnosis (CAD) and AI. By using a vast amount of anatomical, morphological and connectivity information, AI and CAD can significantly help neuroradiologists and neurosurgeons to make effective and efficient diagnoses, accelerating the triage and hence the workflow to initiate the treatment, reducing the human labour as well as the costs [45]. Multiple studies have demonstrated that door-to-needle times play a crucial role in reducing mortality and improving the prognosis, and obtaining and interpreting radiological images, and the lack of access to neurologists are the major reasons causing delays in delivering emergency treatments for conditions such as stroke [46]–[48]. AI can be used to accelerate brain imaging acquisition and interpretation that can be crucial in assisting clinicians in improving the accuracy of their diagnosis [49],[50]. Additionally, AI can be used independently for automatic classification of epilepsy type with 60% AI accuracy compared with 62% clinician accuracy [51], predicting tumour type with 86% AI accuracy [52], predicting glioma with 86% accuracy [53], diagnosing acute ischaemic events with 56% accuracy [54], and cerebral aneurysms with more than 90% accuracy [55].

In pre-operative planning, AI algorithms have been used for automatic tumour segmentation [41],[56], epileptogenic zone localisation [51], selecting appropriate candidates for epileptic surgery [57], predicting symptomatic cerebral vasospasm after aneurysmal subarachnoid haemorrhage [58], and predicting tissue damage following acute ischaemic stroke [59]. For example, classification of epilepsy and tumour can be subjective [50],[60], therefore causing differences in the decision-making of neurosurgeons. By providing a robust framework and outline, algorithms using AI can reduce the subjective interpretation of the data and thereby diagnose conditions requiring neurosurgical procedures.

Effective and efficient identification of glioma tissue is a crucial step in pre-operative planning and can be dependent on the anatomical knowledge of neuroradiologists and neurosurgeons, and therefore, time-consuming and dependent on the operator variation [61]–[64]. While glioma resection can extend patients' survival, it can have high postoperative risk because gliomas can be found in functional areas which control various functions such as sensory, motor, vision and language [65]. AI was successfully used for MRI-based segmentation of glioma and brainstem tumours and did similarly or outperformed physicians [41],[56],[66].

Classification is another task that can be performed by ML. For example, lumbar disk degeneration can be classified using MRI scans from healthy to severely abnormal disks [67]. ML can cluster patients suffering from osteoporotic vertebral fracture based on their pain progression [68], thereby helping their management. ML helped diagnosing paediatric posterior fossa tumours by categorising them into the primitive neuroectodermal tumour, astrocytoma, or ependymoma with 72% accuracy compared to 73% accuracy of neuroradiologists [69] and in other cases with better accuracy than the neuroradiologists [64], as well as classifying intra-axial cerebral tumours including high- and low-grade gliomas, metastatic tumours and malignant lymphomas and sellar-suprasellar masses by significantly increasing the diagnostic classification of tumours by radiologists [50],[70]. Other studies showed that ML and artificial neural network predicted the glioma according to the World Health Organization (WHO) grade better than radiologists [41],[71]–[73]. Beyond tumour diagnosis, ML outperformed physicians with 82.2% to 62.2% accuracy in predicting the presence of abnormal features in CT scans of paediatric TBI patients [74].

AI outperformed physicians (95.8% compared to 66.7%) for lateralising the affected brain hemisphere in the most common pharmacoresistant and surgically remediable type of epilepsy in adults, temporal lobe epilepsy (TLE), using functional MRI data [75]. This can significantly increase the patient outcome as unclear localisation of the epileptogenic zone can be a significant barrier to allocating eligible patients to appropriate surgeries [76].

There are other examples where AI was used in the diagnosis and classification of neurosurgical diseases without radiological input [74],[77]. For example, AI showed significantly higher accuracy in discriminating between single cells vs multiunit spike clusters from electroencephalography recordings of 12 epilepsy patients requiring implantation of chronic intracranial depth electrodes [77]. Since AI is capable of using multiple variables simultaneously, an attribute that is beyond the capacity of a human operator, it can consider multiple factors when planning the treatment. To this end, a study generated an artificial neural network with 11 clinical inputs to train the algorithm for predicting the survival of TBI patients [78]. ML had a better performance in accuracy and sensitivity and was more specific compared to neurosurgeons and neurosurgery residents [78].

AI can predict the progression of a disease. For example, MRI data from a large, multiinstitutional dataset was used to train deep learning algorithms to replace invasive tissue sampling and predict the progression of glioma noninvasively [79],[80], demonstrating the potential of ML in enhancing capitalisation of the existing data.

The current conventional workflow of intraoperative tissue biopsy where the tissue is being transported to a laboratory, it is processed, and specimen are prepared by highly skilled laboratory personnel before being interpreted by pathologists [81] is time-, labour-and resource-intensive and dates back more than a century old [82]. To provide an example of the use of AI in the intra-operative phase of neurosurgery, Hollon et al. [81] have developed a label-free optical imaging workflow to automatically predict diagnosis in near real-time. Their tumour diagnosis methods can predict the diagnosis of tumours in under 150 seconds compared to conventional methods described earlier, which can take 30 minutes [83], with an overall accuracy of 95%, which slightly outperformed conventional histology workflow with an accuracy of 94% [81].

Since patients might require multiple visits to various geographical locations such as outpatient clinics, inpatient wards, pharmacies, emergency departments, intensive care units, and laboratories, telemedicine can reduce the unnecessary travel by both healthcare professionals and patients [84],[85]. Telemedicine is positively valued by patients and healthcare providers and can improve patient outcomes during postoperative care, especially in geographically restricted areas [86]–[88]. Postoperative videoconferencing was welcomed by the majority of patients and was comparably as safe and as effective compared with in-person clinic visits among patients who had elective neurosurgery [87]. As such, telemedicine can be used for follow-up visits which would have required in-person clinic visits, remote diagnosis of postsurgery complications, as well as continued routine monitoring [89]. In comparison, the use of AI in postoperative care and follow-up of neurosurgery is less agreeable among surgeons and surgical teams compared to other usages such as pre-operative planning and image interpretation [90].

These examples clearly demonstrate the power of AI, ML and DL in pre-, intra- and postoperative phases of neurosurgical diseases. Future work requires integration of AI and ML models to combine pre-, intra- and postoperative algorithms into a single model where the best pre-operative planning, intra-operative surgical intervention, and postoperative follow-up work with associated risk, financial cost, and considerations can be suggested to surgeons. Such algorithms not only can benefit neurosurgeons in their decision-making but also facilitate the delivery of high-quality healthcare to low resources settings and facilitate personalised surgical and postsurgical care.

## 4. Expanding access to high-quality neurosurgical healthcare

A number of efforts, such as increasing the capacity of neurosurgical training, can address the disparity associated between the demand and the availability of neurosurgical care discussed earlier in this review; however, such disparities in healthcare still exist.

The COVID-19 pandemic demonstrated the vulnerability of the healthcare systems around the globe. Elective neurosurgeries were cancelled to free up staff and beds for the critical care of pulmonary COVID-19 patients, in addition to a reduced capacity in teaching and training neurosurgery [91]. One of the important lessons that can be learnt to strengthen the healthcare system is the development of telemedicine and teleoperating by incorporating AI in neurosurgery. Robotic surgery with the help of AI can be used to facilitate patient management and surgical operation during the time of increased demand on the healthcare systems such as pandemics [92]. However, the use of teleoperating and telesurgery should not remain restricted to exceptional circumstances, such as infectious diseases pandemic, and it can be extended to deliver high-quality surgical procedures to rural and less accessible areas, battlefields and difficult terrains. Delivery of surgical interventions for patients in situ can minimise the risk and cost associated with patient transfer to large medical centres and decentralising resources available.

There is a clear global disparity in the availability of neurosurgical care, which can result in preventable disease, disability, and death. For instance, while around 7500 neurosurgeons in Japan take care of the neurosurgical needs of the population, it has been estimated that thirty-nine countries around the globe do not have a single practising neurosurgeon [17]. In some low- and middle-income countries, patients must travel in excess of 2 hours to receive emergency neurosurgical care, which itself has a lower quality of care compared to high-income countries [93]. Teleneurosurgery, with the help of AI, can address some of the existing disparities in accessing high-quality healthcare.

Smartphone apps can provide patients with easy access to healthcare providers as well as helping people with behavioural changes to educate patients to improve their general health and well-being and help them monitor their health such as weight management, smoke cessation [84]. Such mobile phone apps can be used for postoperative neurosurgical follow-up. Indeed, AI can be further used to develop mobile phones app for the early detection of other diseases and conditions of the nervous system, such as stress, anxiety and depression, among specific and targeted populations [94] to provide a better prognosis for patients.

Although doctors try to deliver the best possible treatment for their patients, access to high-quality medical care can be restrained by economic means [95], resulting in disparities in access to appropriate healthcare. AI, telemedicine, teleoperating and telesurgery can help ameliorate some of these disparities.

## 5. Using AI to push the boundaries of neurosurgical research

**Figure 5.**
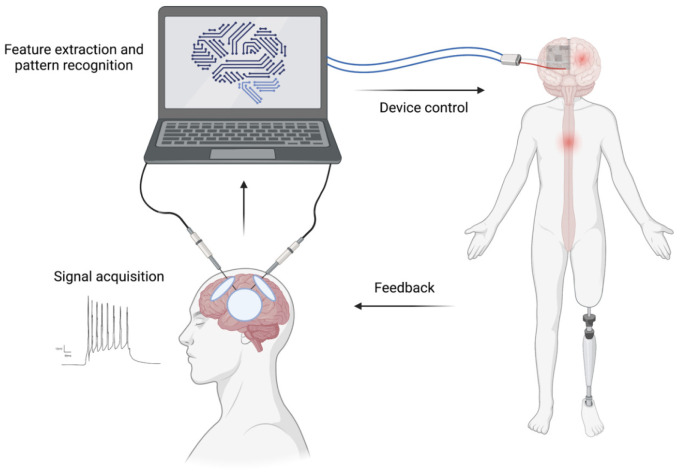
An overview of brain-computer interface (BCI) and AI working together to restore and enhance the sensory and motor functions of the central and peripheral nervous systems. AI can improve BCI by facilitating audio sensation, somatic sensation, visual sensation, and etc. Microelectrodes can pick up the signal from the brain and transfer them to AI for processing. AI can process the signal and extract meaningful features from them, for example, remove the background noise from the readings, identify the logic in the data and produce a coherent outcome [98],[100]. Feedback from the outcome can then be sent to the cortex to adjust the function, thereby providing a real-time adjustment of the behaviour. The figure was made using Biorender.

We live in an era that has been named the age of information [96]. One of the major challenges associated with the age of information is the processing and storage of such a vast amount of clinical data that was briefly discussed before. AI and artificial neuronal network (Figure 5) can be useful tools to helps us comprehend the complexity of the nervous system. With the arrival of more data processing power and accumulation of data, AI has become more successful in surgical research in the past decade. Technology is beginning to demystify the notion of “mind control”. Brain-computer interface (BCI) and AI can be combined to restore some of the sensory and motor functions of patients with paralysis, expand the motor ability of healthy people and facilitate the development of next-generation robots [97],[98]. Currently, about 169,000 people live in the United States of America with traumatic spinal cord injury causing tetraplegia [99]. In these patients, the cortex still generates neuronal activity for limb movement, but the firing is not passed to the limbs due to spinal injuries. Signals can be acquired noninvasively from the scalp using electroencephalography (EEG) and processed to control robotic arms [97].

AI and ML can understand the firing of the cortex, and restore motor control by transmitting signals from the human motor cortex to muscles of a quadriplegic person with a C5/C6 cervical spinal cord injury, thereby providing a “neural bypass” [101]. A recent bidirectional BCI, which provides patients with tetraplegia with substantially improved control over the robotic arm by recording neural activity from the cortex and generating tactile sensation through intracortical stimulation of the somatosensory cortex [102], can have a substantial impact on the advancement of BCI and AI in neurosurgical treatment. Such combinations of AI and BCI had the advantage of providing a real-time adjustment for the desired task as the algorithms can learn from the past experience and help the users to an optimal outcome based on the previous task [103].

As AI becomes more prominent in neurosurgery, it can develop a mutual relationship where AI helps neurosurgery and vice versa. AI and ML have enabled data to speak for themselves and guide physicians to make better decisions [14],[26]. Furthermore, AI can help building and testing hypotheses as well as analysing clinical and experimental data. This frees up clinical researchers' valuable time from carrying out mundane analysis and help them focus on the bigger picture [26]. Furthermore, the accuracy of data analysis can be enhanced by minimising human error and bias [3].

## 6. Challenges associated with using AI in neurosurgery

Using AI in neurosurgery is not completely harmless. There can be negative primary and secondary consequences associated with over-reliance on AI in neurosurgery. At the primary level, hardware and software malfunctions can cause errors in the surgical procedures [6], as well as misinterpretations of clinical findings, laboratory reports, and image scans leading to mistakes in diagnosis. At the secondary level, over-reliance on AI for surgical interventions can discourage surgeons from learning skills required to master surgical techniques. Over-reliance on algorithms for the diagnosis and treatment of nervous system diseases can be problematic. For example, algorithms used in ML can overfit the data [22] to produce false positives or false negatives. However, such malfunctions and malperformance can be ameliorated by confirming the functionality of the algorithm using validation samples and independent data sets [22].

One of the concerns associated with the use of AI in the medical field has been that it will replace clinicians. It is of paramount importance to bear in mind that the patient is at the centre of medicine and benefit to patient should be the most important criterion in deciding whether AI can benefit medicine or not, AI should not replace the human, but it should work cooperatively with surgeons to complement their skills and improve their performance to provide the best possible care. Recent qualitative studies have shown that while patients and their relatives welcome the use of AI in neurosurgery and find it acceptable, they were reluctant to be treated by fully autonomous neurosurgeries and would like to have neurosurgeons to ultimately remain in control [104]. Similarly, most neurosurgeons welcome the use of AI in neurosurgery [90], so there is a consensus among neurosurgeons and patients on the use of AI in neurosurgery. Multiple studies have shown that the combination of AI with clinicians improved clinical decision making compared to clinicians or AI alone [50],[70],[105],[106]. As such, neurosurgeons will not be deskilled, but they can expand on their expertise to equip themselves with better techniques.

Another problem with the use of AI, and especially BCI, is that it is still very limited, and many practical challenges need to be overcome before the widespread usage of AI in restorative neurosurgery. Multiple challenges exist with respect to implant technology, implant recipients and implantation methodologies [107]. Furthermore, generating and training algorithms requires a vast amount of data [31]. This can be further complicated, given that access to most of the patient data is restricted due to privacy concerns [26]. ML is based on data acquisition and availability and any algorithm is as good as the data used to train it. Therefore, successful acquisition and implementation of data are essential for developing and training ML algorithms. Therefore, noisy and complicated data can itself pose a challenge to develop optimal AI algorithm.

Another challenge with the usage of AI in neurosurgery is the cost. However, while the initial training and operating of AI might have some costs, the long-term benefits of reducing surgeons' workload, increasing the handling of data, and reducing error can outweigh the initial cost. Furthermore, ML can reduce the cost associated with unnecessary testing and defensive medicine. However, this requires a better definition for different diseases and conditions.

Furthermore, the storage of a vast amount of data about our neuronal activity can provide abusers with a means to access information about conscious and subconscious behaviours, intentions, desires, and interests which can be used to manipulate human's behaviour [108], indicating development of ethical regulations in the usage of AI in neurosurgery [109].

## 7. Conclusion and outlook

AI is an interdisciplinary field in the interface of medicine, neuroscience, and engineering. Neurosurgery can harvest the power of AI to provide patients with the best outcome. AI has the capacity to improve surgeons' skill set in the pre-, intra- and postoperative arena in neurosurgery. Humans and machines can work cooperatively to harness the recent technological advances in AI to enhance the quality of healthcare delivery through image acquisition, processing and interpretation, allocating patients to appropriate surgeries, improving intra-operative work, providing postoperative follow-up and facilitating access to high-quality healthcare. The use of AI can be further expanded for neuromuscular and neurodegenerative diseases to treat conditions such as Parkinson's disease, which are currently treated by medication and deep brain stimulation [110], as well as understanding different aspects of molecular cell biology such as subcellular trafficking of cargoes in single neurons [111],[112].

The emergence of AI in neurosurgery requires careful regulation and monitoring to abide by ethical principles [109]. The use of AI in medical and surgical training should be incorporated early in undergraduate medical school to train the future generation of surgeons with the state of art technologies. Undergraduate medical students have an awareness of the potential of AI in medicine and are not concerned that AI will substitute human radiologists [113].

AI can be a gateway for personalised medicine in the future. Furthermore, as the acquisition of large clinical data is growing, precision medicine aims to establish quantitative models to predict the health outcome, prognosticate disease procedure, prevent diseases and minimise surgical complications [38]. Both personalised medicine and precision medicine can benefit from AI. Future widespread use of AI in neurosurgery requires further research, investment, and multidisciplinary collaborations.
